# Personal Resources and Organizational Outcomes: Sex as a Moderator of the Complex Relationships Between Self-Esteem, Heart Rate Variability, and Work-Related Exhaustion

**DOI:** 10.3389/fnins.2021.615363

**Published:** 2021-10-05

**Authors:** Evelina De Longis, Cristina Ottaviani, Guido Alessandri

**Affiliations:** ^1^Department of Psychology, Sapienza University of Rome, Rome, Italy; ^2^IRCCS Santa Lucia Foundation, Rome, Italy

**Keywords:** self-esteem (SE), HRV (heart rate variability), exhaustion, negative affect, sex

## Abstract

Global self-esteem represents a protective personal resource lowering the risk of psychological distress. Research conducted in the work setting has confirmed the psychosocial benefits of high self-esteem. However, research linking self-esteem to neurobiological adaptability appears quite scarce. In this study, we propose a theoretical model in which self-esteem predicts work-related exhaustion indirectly, through the mediation of heart rate variability (HRV) and negative affect at work. Moreover, we explore the relationship between self-esteem and HRV. From one side, one would expect a positive link between self-esteem and HRV, signaling higher autonomic adaptability. However, recent studies have shown that in women, such associations become more complex, with even reversed patterns as compared with that in men. Thus, we included sex as a moderator of the relationship between HRV and self-esteem. The model was tested on a sample of 110 individuals working in the relational professions (54% males; M_*age*_ = 42.6, SD = 13.73), observed for an entire workday. Results confirmed the protective role of self-esteem against the experience of negative affect and (indirectly) work-related exhaustion. Symptoms of exhaustion at work were also negatively predicted by HRV, and both HRV and negative affect acted as mediators of the relationship between self-esteem and work-related exhaustion. Notably, sex differences emerged in the association between global self-esteem and cardiac vagal tone at work: in women, self-esteem was negatively related to HRV, which in turn led to higher work-related exhaustion, whereas in men, no evidence of this indirect effect appeared. Burnout prevention programs should not ignore important sex differences in how individuals respond to work-related stress.

## Introduction

Exhaustion is the core component of job burnout syndrome, and it refers to a feeling of depletion of emotional, physical, and cognitive resources (Hobfoll and Shirom, 2000; Maslach et al., 2001). A large body of research documents that exhaustion often results in a number of negative outcomes both for employees and organizations (e.g., Bakker et al., 2014; Bakker and Costa, 2014). Exhaustion, in fact, is associated with psychological and physical health problems, such as cardiovascular diseases, musculoskeletal disorders, pain experiences, depressive and insomnia symptoms, and mental disorders (for a review, see Bakker et al., 2014; Salvagioni et al., 2017). As for its occupational consequences, exhaustion is related to job dissatisfaction, overload, absenteeism, presenteeism, and reduced job performance (both at the individual and at the team level; see Bakker et al., 2014; Salvagioni et al., 2017). Given its significant impact on individuals’ health and well-being, researchers have widely investigated exhaustion’s antecedents in order to better understand and prevent this phenomenon. In this regard, a large body of literature has highlighted the role of individual differences in the development of exhaustion symptoms (for a meta-analysis, see Alarcon et al., 2009). Among these, consistent evidence points to the protective role played by self-esteem (e.g., Hobfoll and Freedy, 1993; Janssen et al., 1999; Alarcon et al., 2009; Alessandri et al., 2017), defined as a global subjective judgment of personal worth and self-acceptance (Rosenberg, 1965; Marsh and O’Mara, 2008). Individuals with high self-esteem are usually more satisfied with their life and more optimistic, have a clearer self-concept, are more likely to experience positive emotions, and have a high sense of mastery (Lyubomirsky et al., 2006). In addition, they are less vulnerable to anxiety and depression (Lyubomirsky et al., 2006). In the workplace, self-esteem proved to be a valuable resource, as it was found to be associated with career success, better working conditions and outcomes, better quality of relationships with colleagues, and low levels of exhaustion (e.g., Janssen et al., 1999; Kammeyer-Mueller et al., 2008; Kuster et al., 2013; Orth and Robins, 2013; Perinelli et al., 2021).

Importantly, under the theoretical framework of conservation of resources (COR) theory (Hobfoll, 1989), self-esteem has been conceptualized as a key personal resource that may help individuals to cope with stressful working conditions, as it affects how they react to an actual resource loss or to the threat of a loss. The main tenet of COR theory is that individuals strive to obtain, retain, and foster their resources (Hobfoll, 1989). It follows that stress occurs when individuals lose resources, are threatened with resource loss, or fail to gain resources after an investment (Hobfoll, 1989). Under this theoretical framework, high global self-esteem can be viewed as a “reserve” of self-worth and confidence that influences individuals’ ability to cope with a (threat of) resource loss (Grandey and Cropanzano, 1999; Hobfoll, 2010; Alessandri et al., 2017). However, despite the evidence of a negative association between self-esteem and work-related exhaustion, the process underlying this relationship has not yet been fully clarified (e.g., Janssen et al., 1999). At the same time, while the benefits of self-esteem for work adjustment are well acknowledged, relatively few studies have explored its physiological correlates (Martens et al., 2008, 2010; Schwerdtfeger and Scheel, 2012), especially in the work setting.

Some evidence is available of the relationship between self-esteem and biological measures. For example, Pruessner et al. (2005) showed that self-esteem is associated with cortisol responses to a psychosocial stress task as well as with hippocampal volume, supporting the notion that perceiving a situation as more stressful, and therefore activating the hypothalamic–pituitary–adrenal (HPA) axis, might have an effect on specific brain structures via the neurotoxic effects of cortisol. Self-esteem has also been found to modulate neural responses to evaluative feedback in the ventral anterior cingulate cortex/medial prefrontal cortex (mPFC) (Somerville et al., 2010), and it has been linked to alpha asymmetry at the precuneus, which plays a key role in self-referential thought (Alessandri et al., 2015). Of interest, a connection between self-esteem and cardiac vagal tone has been proposed (e.g., Martens et al., 2008; Schwerdtfeger and Scheel, 2012). Specifically, some evidence is available for a positive association of self-esteem with heart rate (HR) variability (HRV; e.g., Martens et al., 2008, 2010; Schwerdtfeger and Scheel, 2012), a measure of parasympathetic modulation of the heart and measured as the variability in time between successive heart beats (Task Force, 1996). For instance, Martens et al. (2008, 2010), moving from the assumption that high self-esteem and high HRV represent resources that buffer threat responses, conducted four studies on state-dependent changes in self-esteem and their association with resting HRV. These authors found that positive self-esteem relevant feedback (i.e., bogus personality feedback) was related to higher HRV compared with negative feedback, even when controlling for negative and positive mood (Martens et al., 2010, Study 1). Furthermore, the authors found that a positive intelligence feedback was associated with an increase in HRV relative to a negative intelligence feedback (Martens et al., 2010, Study 2) and that overall levels of self-esteem over 2 weeks were positively correlated with resting HRV assessed in the laboratory (Martens et al., 2010, Studies 3 and 4). Relatedly, O’Donnell et al. (2008) found that greater global self-esteem was associated with lower HRV during a speech task. Of special interest for the present study, Schwerdtfeger and Scheel (2012), using ecological momentary assessment among college students, found evidence for a positive association between self-esteem and HRV in men, while such relationship was negative, although not significant, in women. The authors also reported a negative relationship between self-esteem and negative affect, which was considerably stronger in women than in men (Schwerdtfeger and Scheel, 2012).

Taken together, these findings are in line with the neurovisceral integration model (Thayer and Lane, 2000, 2009), which suggests that HRV can be used as an index of physiological, emotional, cognitive, and behavioral processes involved in self-regulation and adaptability. In the work setting, evidence is accumulating on the negative relationship between HRV and work-related exhaustion (Lennartsson et al., 2016; Kanthak et al., 2017; De Longis et al., 2020), as well as negative emotions at work (Pieper et al., 2007; Uusitalo et al., 2011; De Longis et al., 2020).

**FIGURE 1:**
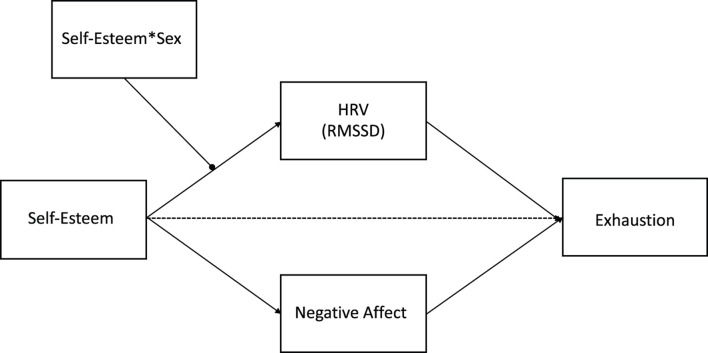
Conceptual representation of the hypothesized moderated mediation model. Self-esteem*Sex = interaction term between self-esteem and sex.

On the basis of these findings and the theoretical arguments derived from COR theory (Hobfoll, 1989; Orth and Robins, 2013; Burić et al., 2019), the present study aims to extend previous research by examining the contribution of HRV to the health-protective effect of self-esteem in the workplace. To this end, we used the experience sampling method combined with HRV assessment to test a comprehensive model (see Figure 1), which takes into account the role of both physiological and psychological variables in the association between self-esteem and work-related exhaustion. Furthermore, in light of recent evidence of substantial sex differences in vagal activity, we also tested the moderating effect of sex in the relationship between self-esteem and HRV (e.g., Snieder et al., 2007; Koenig and Thayer, 2016; Jarczok et al., 2018). More in details, in line with previous studies (e.g., Janssen et al., 1999; Alarcon et al., 2009; Orth and Robins, 2013) and with COR theory tenets, we expect a negative relationship between workers’ self-esteem and work-related exhaustion, and we expect HRV and negative affect to act as mediators of this relationship. Self-esteem, in fact, has been found to represent a good proxy measure for well-being (e.g., Diener et al., 1999) and to be related with emotion regulation and emotional experiences (e.g., Nezlek, 2005; Lightsey et al., 2006; Nezlek and Kuppens, 2008). In addition, it seems reasonable to expect that self-esteem, as a coping-enhancing factor, may be related to lower levels of negative emotions and high HRV (e.g., Hughes, 2007). Stated differently, two parallel processes are expected to underlie the relationship between self-esteem and work-related exhaustion. On the one hand, from a psychological point of view, negative affect may act as a mediator of the relationship between self-esteem and work-related exhaustion: those high in self-esteem, and being less prone to experience negative emotions at work, may report lower exhaustion. On the other hand, from a physiological point of view, HRV is also postulated to mediate the negative relationship between self-esteem and work-related exhaustion: high self-esteem, by increasing cardiac vagal tone at work, may lead to reduced exhaustion symptoms. In light of the well-documented sex differences in both the neural control of HRV, with higher HRV in women compared with man (e.g., Koenig and Thayer, 2016; Tobaldini et al., 2020) and in self-esteem (Kling et al., 1999; Zuckerman et al., 2016), we also aimed at exploring the interactive effect of sex and self-esteem on HRV at work. To our knowledge, only one previous study examined the role of sex in the association between self-esteem and HRV. In the study conducted by Schwerdtfeger and Scheel (2012), self-esteem fluctuations were significantly positively associated with HRV for men and were negatively, but not significantly associated with HRV for women, suggesting a sex-specific relationship between self-esteem and HRV.

We tested our model with a sample of workers observed for an entire working day, during working hours. Several studies, indeed, showed that the psychosocial work environment influences HRV (for a review, see Jarczok et al., 2013). Finally, following methodological guidelines (e.g., Quintana et al., 2016), in testing our model, we controlled for body mass index (BMI), age, medications, caffeine, posture, physical effort, and nicotine consumption.

## Materials and Methods

### Participants

Our sample consisted of 110 individuals working in the relational professions. Most of them (54.2%) were males, with a mean age of 42.6 years (*SD* = 13.73). They had an average BMI of 24.02 (*SD* = 3.63); 27.7% of participants were smokers, and 77.5% of participants did not take any medication that could affect HRV. A diagnosis of heart disease was an exclusionary criterion. In terms of employment sectors, 24.8% of participants worked in the sales sector, 10.3% of participants worked in the health sector, 8.5% of participants worked in the education sector, 10.8% were entrepreneurs, 9.8% of participants were managers, and the remaining 35.8% were employees in various fields. The mean job tenure was 14.5 years (*SD* = 11).

### Procedure

Participants were recruited mainly through online advertisement, but also via word of mouth. A week before the experience sampling procedure, socio-demographic information and global self-esteem were assessed in an initial online survey. All participants also provided informed consent, as well as their work schedule for the day of the study, indicating their start and end times and breaks at work. Then, in the selected day of the following week, participants were prompted semi-randomly (via a tone signal on their smartphone) six times during working hours. The time interval between two assessments varied depending on the length of the workday (e.g., for a 6-h workday, prompts occurred semi-randomly about every 60 min). Depending on the length of the workday, participants had 10 or 20 min to respond to the initial question (e.g., for a 6-h workday, participants had 10 min to fill in a questionnaire; for an 8-h workday, participants had 20 min to fill in a questionnaire).

### Measures

#### Self-Esteem

To measure global self-esteem, we used the Rosenberg Self-Esteem Scale (RSE; Rosenberg, 1965). A sample item is “I feel that I have a number of good qualities.” Each of the 10 items was scored on a 4-point scale ranging from 1 = “Strongly disagree” to 4 = “Strongly agree.” Alpha coefficient was 0.77.

#### Heart Rate

HR was monitored using Bodyguard 2 (Firstbeat) HR monitors, which have been extensively used for HR recording and provide reliable measures of beat-to-beat intervals (e.g., Porto and Junqueira, 2009). Participants were instructed to wear the HR monitor for 24 consecutive hours on the selected day (to include the entire work shift and one night of sleep). As a first step, each of the six surveys entries was labeled in the cardiac data. Then, we divided the 24-h raw beat-to-beat intervals in several blocks, one for each interval between two prompts. We removed from the analysis any break from work (e.g., lunch break). To assess vagally mediated HRV, we computed the root mean square of successive beat-to-beat interval differences (RMSSD), the recommended parameter for field studies, as it reflects vagal regulation of HR being less affected by breathing (Task Force, 1996; Penttilä et al., 2001; Laborde et al., 2017). HRV analyses, outlier, and artifact detection were performed using Kubios HRV software (Tarvainen et al., 2014).

#### Negative Affect

To measure negative affect, we asked participants to report their current levels of negative emotions. In each survey, participants indicated the extent to which they were currently feeling each of nine negative emotions (sad, angry, anxious, ashamed, frustrated, irritable, guilty, restless, and disgusted) by moving a slider anchored with the numbers 0 and 100. The selected affect words were drawn from the study by Thompson et al. (2012) and De Longis et al. (2020). The between-person reliability, calculated by running mixed models and following the procedure indicated by Bonito et al. (2012), was 0.40, while the within-person reliability was 0.86. The within-person value can be considered substantial (Shrout, 1998). In the present study, an aggregated value of negative affect, representing the observed mean level of negative affect observed across the six prompts that occurred during the workday, was used.

#### Exhaustion

At the end of the workday, work-related exhaustion was assessed by using two items drawn from the Maslach Burnout Inventory—General Survey (MBI-GS; Maslach et al., 1996; i.e., “I feel emotionally drained from my work” and “I feel exhausted by my work”). The items were scored on a 5-point scale (1 = very little or not at all; 5 = extremely). Alpha coefficient was 0.82.

### Statistical Analysis

Our model was tested within the framework of Bayesian structural equation modeling (BSEM), using Mplus 8 (Muthén and Muthén, 2018). BSEM fit was assessed according to the following criteria: the posterior predictive *p*-value and the associated 95% credibility interval (Muthén and Asparouhov, 2012). Credibility intervals are the Bayesian analogous of frequentist confidence intervals, from which they differ as they are based on the percentiles computed on the whole distribution of the posterior estimates. In addition, the derivation of these intervals does not rely on large-sample theory and does not assume that the distribution is normal. A good model fit is expected to show a posterior predictive *p*-value of approximately 0.5 and a symmetric 95% credibility interval (CI 95%) centered around zero. All parameter estimates with an associated posterior *p*-value value below 0.05 were considered statistically significant (*p* < 0.05).

## Results

### Descriptive Statistics and Correlations

Descriptive statistics and correlations among the main study variables are reported in Table 1. Except for HRV, all variables were significantly correlated in the expected direction. In the overall sample, moderate correlations were found among work-related exhaustion and negative affect, as well as negative affect and self-esteem. HRV was negatively and significantly related only to work-related exhaustion.

**TABLE 1:** Means, standard deviations (SD), and correlations among variables.

Overall sample		Mean	*SD*	1	2	3	4
	1. Self-esteem	3.28	0.43	1	–	–	–
	2. Negative affect	8.44	10.73	−0.33**	1		–
	3. lnHRV	1.46	0.27	−0.14	−0.07	1	–
	4. Exhaustion	1.76	0.88	−0.31**	−0.39**	−0.19*	1
	5. Sex	–	–	0.13	−0.18*	0.03	0.00

**Stratified by sex**		**Mean_*w*_ (SD_*w)*_**	**Mean_*m*_ (SD_*m)*_**	**1**	**2**	**3**	**4**

	1. Self-esteem	1.78 (0.47)	1.67 (0.38)	1	−0.25*	0.00	−0.36**
	2. Negative affect	10.59 (12.47)	6.70 (8.81)	−0.37**	1	−0.18	0.39**
	3. lnHRV	1.45 (0.25)	1.47 (0.28)	−0.31*	0.05	1	−0.26**
	4. Exhaustion	1.76 (0.81)	1.77 (0.94)	−0.26	0.43**	−0.09	1

Means for daily hassles and exhaustion were aggregated within and across days. Correlations for women are presented below the diagonal; correlations for men are presented above the diagonal. lnHRV = natural logarithm of heart rate variability; w = women; m = men. *p < 0.01. **p < 0.05.

Table 1 also presents descriptive statistics and correlations stratified by sex. Interestingly, self-esteem was negatively correlated with HRV in women, whereas there was no observed correlation in men. Exhaustion was negatively and significantly associated with HRV only in men. The negative correlation between self-esteem and exhaustion was statistically significant only in men.

### Model Testing

**FIGURE 2:**
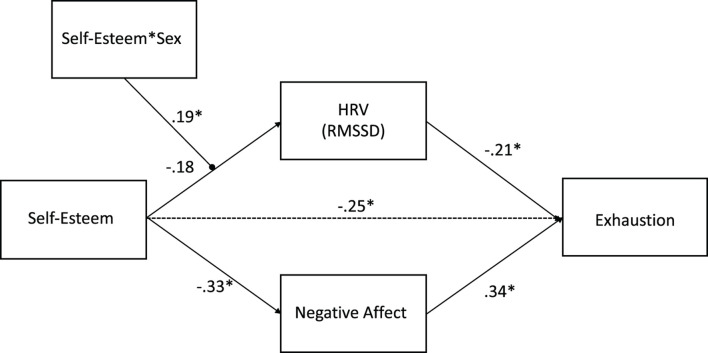
The moderated mediation model with parameter estimates. Self-esteem*Sex = interaction term between self-esteem and sex.

**FIGURE 3:**
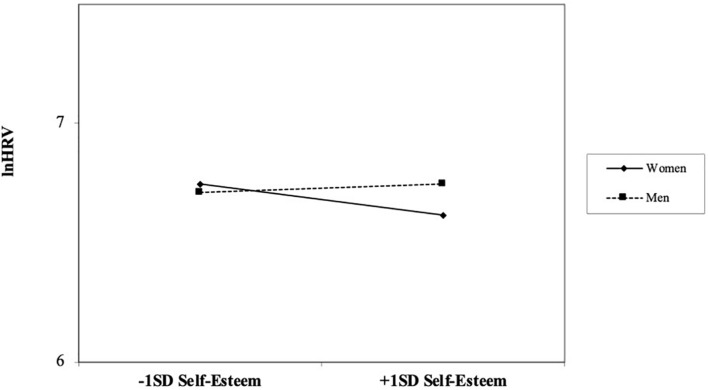
Interaction of self-esteem and sex on heart rate variability (HRV).

The hypothesized model fitted the data well, as indicated by a posterior predictive *p*-value of 0.19 and a symmetric 95% posterior predictive interval ranging from −13.315 to 33.197. This model is shown in Figure 2. As it can be seen, the relation between self-esteem and HRV was qualified by a significant interaction between self-esteem and sex. HRV, on the other hand, significantly and negatively predicted work-related exhaustion. At the same time, self-esteem significantly and negatively predicted negative affect, and negative affect significantly and positively predicted work-related exhaustion. Thus, both HRV and negative affect acted as mediators of the relationship between self-esteem and work-related exhaustion. Hence, as a next step, simple slopes at one standard deviation above and below the mean of the scores on self-esteem were computed. The observed relationship between self-esteem and HRV as a function of sex is represented in Figure 3. Results from simple slope analysis indicated that the relationship between self-esteem and HRV was not significant for men (*B* = 0.024, *p* = 0.39), while it was significant and negative for women (*B* = −0.162, *p* = 0.019). Accordingly, women with low self-esteem were characterized by higher HRV, as compared with women with high self-esteem. The latter, on the contrary, were characterized by lower HRV.

The finding of a conjoint prediction (1) of HRV by the interaction term between self-esteem and sex and (2) of exhaustion by HRV suggested that the indirect association between self-esteem and exhaustion might be moderated by sex. In accordance, we found that the indirect effect of self-esteem on exhaustion via HRV was significant and positive only for women (0.12, *p* = 0.02) but was not significant for men (−0.02, *p* = 0.39). Likewise, we found a significant indirect effect of self-esteem on exhaustion symptoms through negative affect (−0.28, *p* = 0.002).

Finally, among covariates, only age was significantly and negatively associated with HRV (−0.01, *p* < 0.001).

## Discussion

The aim of this study was to investigate two possible pathways through which global self-esteem, one of the most popular individual differences constructs in psychology (Donnellan et al., 2011), may protect workers from developing exhaustion symptoms. Despite that self-esteem has been acknowledged as a key personal resource capable of influencing how individuals react to challenging circumstances (Hobfoll, 1989; Janssen et al., 1999), the mechanisms through which it may operate need further investigation. Studies investigating general self-esteem and its association with neurobiological adaptability are relatively scarce and typically involved samples of college students, leaving the relationship between self-esteem and HRV unexplored in the organizational domain.

A first finding of this study is that global self-esteem is associated with lower negative affect at work, thus indirectly protecting workers from exhaustion symptoms. This result is consistent with COR theory conceptualization of self-esteem as a personal resource that dampens threat responses, as it is associated with a feeling of security and confidence that offsets the impact of job stressors and their consequences (e.g., Hobfoll, 1989; Hobfoll and Freedy, 1993). Furthermore, the negative association between self-esteem and work-related exhaustion is consistent with previous research, suggesting an effect of self-esteem on work outcomes (Kuster et al., 2013). The mediating role of negative affect, on the other hand, broadens our understanding of the mechanisms through which an individual difference (i.e., self-esteem) may protect workers from chronic stress conditions. This pattern of results suggests that the reduced perceived vulnerability associated with self-esteem may affect individuals’ affective reactions at work, possibly because it leads to a less threatening interpretation of potentially stressful work events, or because it supports the use of efficient coping strategies.

Symptoms of exhaustion at work were also significantly and negatively predicted by HRV. This result fits well with a plethora of results highlighting the prognostic value of HRV as an indicator of future negative psychological outcomes, for example, daily instability of positive affect (e.g., Koval et al., 2013) or the development of depressive symptoms over time (e.g., Carnevali et al., 2018). The neurovisceral integration model (Thayer and Lane, 2000, 2009) illustrates that this is plausible from a physiological point of view thanks to afferent and efferent vagal pathways, which allow emotional states to affect physiological function and vice versa. According to the model, HRV is a psychophysiological proxy of vagal activity and therefore is a good candidate to reflect good emotion regulation abilities and engagement in context-appropriate behaviors over time.

The second most noteworthy finding, however, concerns the moderating role of sex in the relationship between self-esteem and HRV. Women with high self-esteem, in fact, were characterized by low HRV and, through the mediation of HRV, ended in higher work-related exhaustion than were men. This is not the only paradoxical result that emerged from studies examining HRV in women. Similar to the pattern found between self-esteem and HRV, the association between depressive symptoms and HRV appeared to be positive in female monkeys but not in males (Jarczok et al., 2018). Intriguingly, amygdala activity is negatively correlated with HRV in men but positively correlated in women (Nugent et al., 2011). The amygdala plays a crucial role in adjusting physiological and behavioral responses to stress, for example, exerting strong regulatory influence over the HPA axis and the autonomic nervous system (ANS). When the environment is perceived as safe, amygdala activation is inhibited by the mPFC. Notably, chronic occupational stress is associated with impaired functional connectivity between the amygdala and the mPFC, indicating a reduced capacity to flexibly adjust to the environmental requests (Golkar et al., 2014). A recent review of the literature has reported between-sex differences in the described amygdala–PFC responses to stress (Zhang et al., 2021). Crucially, such differences have been imputed to the role of oxytocin (Bredewold and Veenema, 2018). For example, a psychosocial stressor such as social defeat increases oxytocin production, which in turn regulates output of the vagal dorsal motor nucleus, thereby regulating bodily functions associated with the parasympathetic nervous system, in female mice but not in males (Steinman et al., 2016).

It is therefore clear that there are important sex differences in how individuals respond to stressors. The present study added a further piece in the puzzle showing that the seemingly paradoxical response described above is particularly true for women with lower self-esteem. Importantly, current results replicated in the work setting those of Schwerdtfeger and Scheel (2012), which were obtained by examining a sample of university students. We can speculate that having a higher self-esteem at work comes with a cost for women, as this dispositional characteristic is likely to be associated with an active coping style, while working in the relational professions often requires to suppress and regulate one’s emotions (Thayer et al., 2003). Recent evidence points to the “interpersonal roots” of self-esteem by showing the impact of the interpersonal environment at work on perceived self-worth (Perinelli et al., 2021). It is likely that perceived social support may help to explain variations in individual responses to stressors and their well-being at work, as well as results on HRV in women. Of interest, low social support was found to lead to higher variance in the HRV trajectory, compared with high social support (Baethge et al., 2020). Future studies are certainly needed to clarify the causal mechanisms underlying the role of self-esteem in sex differences in the physiological response to job-related daily stressors.

Overall, findings from this research offer some important contributions to our understanding of the role of self-esteem in workers’ adjustment. First, our study encourages further study of the physiological correlates of global self-esteem, as it sheds light on the pathways between self-esteem and health-related outcomes. Despite that the connection between self-esteem and cardiac vagal tone has been proposed to be context dependent (Martens et al., 2008), this is the first study to investigate such relationship in the work setting, where global self-esteem proved to be a valuable resource that protects against work maladjustment (Kuster et al., 2013).

Results from this study come with some limitations. A first limitation is the use of only two items to assess exhaustion. Given the intensive nature of our study design (six assessments during working hours), we tried to reduce the burden on participants as much as possible and considered two representative items to be sufficient. A second possible limitation concerns the temporal frame considered. Future organizational research should consider the use of repeated assessment of HR over multiple days. Future studies should also control for menstrual cycle phase to better investigate the association between psychological and physiological variables in women. A recent meta-analysis supports the notion that hormonal status may greatly affect such associations (Schmalenberger et al., 2019). Ideally, future research should include multiple physiological indicators other than HRV, such as cortisol, which is intimately linked to psychosocial stress. Finally, it would be desirable to test the generalizability of our findings across different populations of workers.

Notwithstanding these limitations, in this study, we found evidence for sex differences in the association between global self-esteem and cardiac vagal tone at work. While self-esteem was negatively related to HRV in women, in men, such relationship was not significant. Furthermore, both HRV and negative affect acted as mediators of the relationship between self-esteem and work-related exhaustion symptoms at the end of the workday, thus offering insights on the physiological and psychological mechanisms of this relationship and on the prevention of work-related stress.

## Data Availability Statement

The data analyzed in this study is subject to the following licenses/restrictions: data from this study are not publicly available as informed consent and ethical approval for public data sharing were not obtained from participants. Requests to access these datasets should be directed to GA and corresponding author.

## Ethics Statement

The studies involving human participants were reviewed and approved by the Institutional Review Board of Sapienza University of Rome (Prot No. 0000231). The patients/participants provided their written informed consent to participate in this study.

## Author Contributions

GA conceptualized this study. ED and GA collected, prepared the data, and performed the data analysis. ED drafted the manuscript. GA and CO commented and revised the manuscript. All authors contributed to the article and approved the submitted version.

## Conflict of Interest

The authors declare that the research was conducted in the absence of any commercial or financial relationships that could be construed as a potential conflict of interest.

## Publisher’s Note

All claims expressed in this article are solely those of the authors and do not necessarily represent those of their affiliated organizations, or those of the publisher, the editors and the reviewers. Any product that may be evaluated in this article, or claim that may be made by its manufacturer, is not guaranteed or endorsed by the publisher.
